# PIK3CA-related overgrowth spectrum (PROS): a rare case report

**DOI:** 10.1097/MS9.0000000000000485

**Published:** 2023-04-07

**Authors:** Cedra Kalo, Firas Khana, Aya Jazmati, Sima Kalo, Silva Ishkhanian, Alae Kayyali, Taher Sawas

**Affiliations:** Departments of a Dermatology and Venereology; b Medical Imaging and Diagnostic Radiology, Aleppo University Hospital (AUH); c Faculty of Medicine, University of Aleppo, Aleppo, Syria

**Keywords:** case report, CLOVES syndrome, computed tomography angiography, magnetic resonance imaging (MRI), PIK3CA-related overgrowth spectrum (PROS)

## Abstract

Case report.

**Introduction and Importance:**

The purpose of this study is to report an extremely rare case of PIK3CA-related overgrowth spectrum.

**Case Presentation:**

A 12-year-old boy presented with severe overgrowth in the left lower limb causing severe movement restriction and decreased quality of life.

**Interventions and Outcome:**

Episodes of myiasis were manually treated through mechanical removal and the patient was placed on rapamycin therapy for managing vascular malformations.

**Conclusion:**

CLOVES syndrome is a rare overgrowth disorder that can be confused with other overgrowth syndromes; however, clinical and imaging findings are essential for pinpointing the correct diagnosis as genetic sequencing may not always prove reliable.

## Introduction

Highlights

- CLOVES syndrome is an extremely rare genetic overgrowth disorder syndrome.
- CLOVES syndrome is often misdiagnosed as Proteus syndrome (PS).
- Diagnosis is established by relying mainly on clinical and imaging findings.
- Prominent features include lipomatous overgrowth, vascular and skeletal anomalies.
- Rapamycin is a promising drug for the management of vascular malformations.

PIK3CA-related overgrowth spectrum (PROS) is a group of rare genetic asymmetric overgrowth disorder syndromes caused by a mutation in phosphatidylinositol 4,5-bisphosphate 3-kinase catalytic subunit alpha (PIK3CA) gene^1^.

PROS includes CLOVES syndrome, Klippel-Trenaunay-Weber syndrome (KTWS), dysplastic megalencephaly, fibroadipose hyperplasia, hemihyperplasia, multiple lipomatosis, megalencephaly-capillary malformation and macrodactyly. CLOVES syndrome is a nonhereditary disorder with sporadic occurrence characterized by congenital lipomatous overgrowth, skeletal and vascular malformations, epidermal nevi, and spinal anomalies^2^–^5^.

We present the first reported case of PROS in Syria, after scouring the literature in search for similar cases, and based on clinical and radiological findings, we are more likely to suggest CLOVES syndrome as initially described by Sapp *et al.* in 2007^6^, and as later explained by Alomari and colleagues in 2009. The total number of reported CLOVES syndrome cases worldwide is approaching 200 at the time of writing this manuscript^7^.

This case report has been reported in line with the CARE criteria^8^.

## Case presentation

A 12-year-old White boy presented with a 4-year history of gradual overgrowth in his left leg. The pace increased significantly in the past year causing further restriction of the patient’s movements.

Clinical history revealed that the patient was born on-term with a huge congenital lipomatous mass in the back and linear epidermal nevi on the trunk.

No consanguinity was reported between the parents. The mother had an unremarkable history during pregnancy, with no history of smoking or alcohol consumption.

The patient had no history of allergies, and further questioning revealed a single episode of cellulitis and two episodes of cutaneous myiasis occurring in the left lower limb. He also exhibited moderate intellectual disability with learning difficulties.

**Figure 1:**
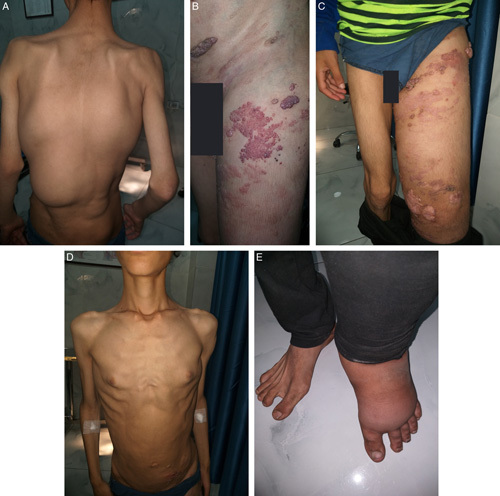
Gross clinical findings. (A) Two congenital lipomatous masses in the trunk, one reaching the left buttock. (B) Severe overgrowth in the left lower limb with leg length discrepancy. (C) Epidermal nevi with capillary malformations. (D) Pectus excavatum deformity in the chest wall. (E) Sandal gap deformity.

Physical examination revealed bilateral lipomatous masses in the lumbar region, with one extending inferiorly to the left gluteal area, as seen in Figure 1A.

There is a severe overgrowth in the left lower limb with a leg-length discrepancy of about 5 cm, accompanied by the presence of linear epidermal nevi on the left side of the trunk, with small asymptomatic bright red macules slowly increasing in size, and tightly coupled translucent vesicles exudating clear colorless fluid, most likely lymph, resembling capillary malformations, as demonstrated in Figures 1B and C, respectively.

The patient also had an inward depression of the sternum and medial ends of the ribs corresponding with the pectus excavatum, as shown in Figure 1D.

**Figure 2:**
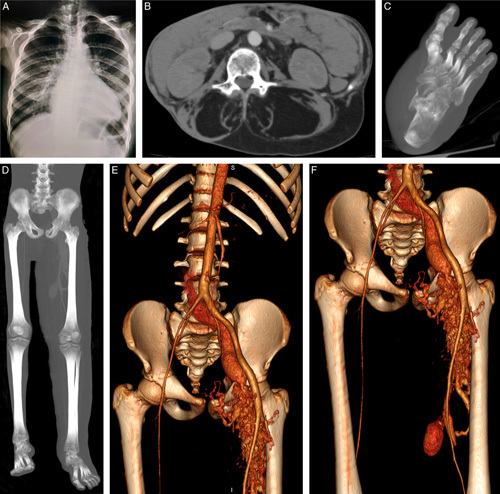
X-ray and contrast-enhanced computed tomography findings. (A) Posteroanterior chest x-ray showing mild lateral scoliosis. (B) Axial view in the arterial phase showcasing two nonenhancing lipomatous masses in the trunk replacing the paraspinal muscles. (C) Maximum intensity projection view of the left foot showing sandal gap deformity with increased soft tissue density between the first and second toes. (D) Maximum intensity projection view of the lower limbs demonstrating the severe overgrowth with leg-length discrepancy of 5 cm. (E and F) Three-dimensional reconstruction of computed tomography angiography in the arterial phase showing arteriovenous fistulas with venous malformations and a superficial femoral vein aneurysm.

An increase in the distance between the first and second toes was observed, suggesting the presence of sandal gap deformity. This is shown in Figure 1E and was later confirmed on computed tomography imaging (Fig. 2C).

Skeletal examination showed a mild lateral curvature of the spine, in line with mild scoliosis, also shown in Figure 1A. This was later confirmed by a posteroanterior chest x-ray, as illustrated in Figure 2A.

Laboratory investigations were unremarkable with normal creatinine and urea levels.

An ECG was ordered, followed by an echocardiogram, and both returned normal.

Ultrasonography of the abdomen was unremarkable, with normal reported dimensions of the liver, spleen, kidneys, and pancreas. No discrepancy was found between the measurements of the kidneys.

Contrast-enhanced computed tomography confirmed the presence of two massive nonenhancing lipomatous overgrowths in the trunk, as depicted in Figure 2B. An increase in the soft tissue component between the first and second toes was also noted, indicative of sandal gap deformity, as shown in Figure 2C. There was a measurable leg-length discrepancy of 5 cm, as seen in Figure 2D. Vascular malformations were present in the form of two high-flow arteriovenous fistulas, one between the left iliac vessels, and the other between the left femoral vessels, as demonstrated in Figures 2E and F, respectively.

**Figure 3:**
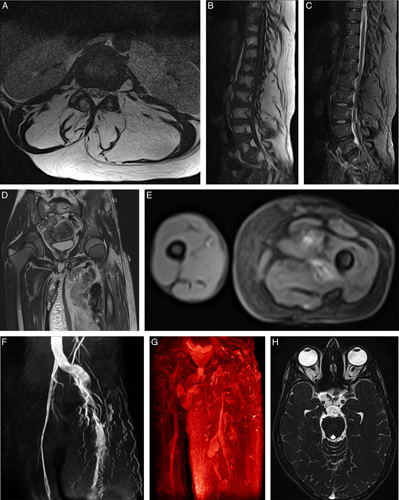
Nonenhanced MRI findings. (A) Axial plane showing two hyperintense lipomatous masses in the truncal area on T2-weighted image. (B and C) Sagittal planes highlight the fatty nature of the masses on T2-weighted and fat-suppressed images. (D and E) Coronal and axial planes demonstrating severe lymphedema and limb overgrowth on T2-weighted images. (F and G) Two-dimensional time-of-flight image showing the arteriovenous fistulas, and a three-dimensional fresh-blood-image using the hot-metal color map showing the venous malformations and the superficial vein aneurysm. (H) Axial plane illustrating the bilateral papilledema on a three-dimensional steady-state-free-precession image.

An MRI of the brain, lumbar spine, and left lower limb was ordered. Two hyperintense lipomatous masses were seen in the lumbar area on T2-weighted images, infiltrating the erector spinae muscles, as shown in Figures 3A and B, along with numerous vertebral hemangiomas. The signal from the lipomatous tissue was properly suppressed on sequences that use fat suppression methods, such as STIR, further confirming its fatty origin, as in Figure 3C. An increase in the soft tissue component was observed in the left lower limb, accompanied by severe lymphedema, denoting the severe overgrowth, as seen in Figures 3D and E. Vascular malformations were best seen on two-dimensional time-of-flight images, and three-dimensional fresh-blood-imaging images, as shown in Figures 3F and G, respectively.

Ocular examinations demonstrated bilateral papilledema with extensive pigment changes. This was also seen on three-dimensional steady-state-free-precession MRIs of the brain, as demonstrated in Figure 3H.

## Discussion

CLOVES syndrome is an extremely rare disorder with a mutation in the PIK3CA gene. Clinical and imaging findings are key to diagnosis as genetic sequencing is not established as a gold standard for diagnosis^9^–^11^.

Compared to other PIK3CA-related overgrowth syndromes, CLOVES syndrome may be confused with and misdiagnosed as PS^9^.

PS is known for the presence of cerebriform connective tissue nevi in association with rapidly progressive postnatal asymmetric bone overgrowth and dysregulated adipose tissue that evolves with disease progression^9^.

In contrast, CLOVES syndrome is characterized by the existence of a lipomatous overgrowth present at birth, particularly in the trunk, with absence of connective tissue nevi, and is accompanied by both high and low-flow vascular abnormalities, both of which were present in our patient and are hardly ever found in PS^9^,^12^.

In our case, the erector spinae muscles were involved in the congenital truncal lipomatous mass, which is in line with the classic presentation of CLOVES syndrome, with numerous vertebral hemangiomas noted in the lumbar region^1^.

Furthermore, gradual swelling of soft tissue is noted in CLOVES syndrome in comparison with Proteus and other syndromes. This swelling, often termed as ‘ballooning,’ can regress by inhibiting the PIK3CA gene mutation that regulates the PI3K/AKT/mTOR pathway^9^,^13^.

KTWS is another common overgrowth disorder marked by the presence of congenital slow-flow vascular malformations (including venous, capillary, and lymphatic) and unilateral hypertrophy in the lower limbs, particularly in the subcutaneous adipose tissue. However, it lacks the lipomatous mass in the trunk, the epidermal nevi, and skeletal malformations. Almost all patients with KTWS have port-wine stains, which is missing in our case^1^,^14^.

Surgical excision or suction-assisted lipectomy remains the first-line treatment for the lipomatous mass, despite having high recurrence rates^14^.

Moreover, CLOVES syndrome is also known for the association of vascular anomalies with skeletal malformations, epidermal nevi, and spinal anomalies. These include sandal gap deformity, polydactyly, macrodactyly, furrowed soles, leg length discrepancy, scoliosis, and pectus excavatum^1^,^5^,^15^,^16^.

Vascular manifestations in CLOVES syndrome include both high-flow and low-flow abnormalities, phlebectasia with capillary malformations covering the overgrowing limb. This finding is critical to the diagnosis of CLOVES syndrome according to Dr. Hermann Friedberg’s reports^1^.

Patients with CLOVES syndrome reported by Alomari *et al.* 1 had high-flow vascular malformations in the thorax with paraspinal lipomatous lesions, while our patient had a truncal lipomatous mass with high-flow arteriovenous fistulas in both the left iliac and femoral vessels. His left leg was also covered with capillary malformations including bright red macules and vesicles filled with clear or turbid colorless fluid suggestive of angiokeratomas and lymphangioma circumscriptum respectively as depicted in the gross clinical images and as later confirmed by histopathological findings^17^.

The patient had suffered one episode of cellulitis and two episodes of cutaneous myiasis in the left lower limb which were treated by manual removal of larvae, and application of topical and systemic antibiotics.

Epidermal nevi were distributed in the left flank along Blaschko lines and were excised under local anesthesia.

CLOVES syndrome can also be associated with seborrheic keratosis, lichenoid keratosis, and several other visceral anomalies including renal hypoplasia, Wilms’ tumor, and gastrointestinal bleeding from vascular malformations^9^. These were luckily not present in our patient.

Some neurological manifestations, albeit uncommon, may be present in CLOVES syndrome. These include polymicrogyria, seizures, tethered cord, hemimegalencephaly, and other neurological impairments^9^. The patient reported in our case had a moderate intellectual disability.

We also report bilateral papilledema accompanied by widespread pigment changes, which is usually found in patients with PS^18^. These findings are rarely described in the current reported cases of CLOVES syndrome, which lead us to believe that they may be quite rare.

Coming up with a treatment approach for CLOVES syndrome patients can be a daunting task. Many reports shed light on rapamycin and its role in suppressing vascular growth in the PROS spectrum. Rapamycin acts by regulating cell growth and inhibiting tumor proliferation, lymphangiogenesis, and vascular permeability^7^,^19^.

Prognosis is unfortunately inconspicuous with the standing risk of developing pulmonary embolism as a complication of the vascular anomalies, if left untreated^11^.

## Conclusions

CLOVES syndrome is an extremely rare disorder that may easily be confused with other overgrowth syndromes^1^,^10^. Many patients with CLOVES syndrome were initially misdiagnosed with PS^20^. The presence of congenital truncal lipomatosis is a strong indicator of CLOVES syndrome and can help exclude other syndromes, including Proteus, where such a sign is absent at birth^1^. Rapamycin has been proven to be effective in the treatment of vascular and lymphatic anomalies^3^.

## Ethical approval

This case report did not require review by the ethics committee.

## Consent

Written informed consent was obtained from the patient for the publication of this case report and accompanying images. A copy of the written consent is available for review by the Editor-in-Chief of this journal.

## Sources of funding

This research did not receive any specific grant from funding agencies in the public, commercial, or not-for-profit sectors.

## Author contribution

C.K. and A.J.: contributed to data collection and interpretation, and writing the paper. F.K.: contributed to data collection and interpretation, study concept and design, and writing the paper. S.K.: contributed to data collection and writing the paper. S.I. and A.K.: contributed to data interpretation and reviewing the paper. T.S.: contributed to data collection and interpretation, and reviewing the paper.

## Conflicts of interest disclosure

The authors declare that they have no financial conflict of interest with regard to the content of this report.

## Research registration unique identifying number (UIN)

Not applicable.

## Guarantor

Firas Khana.

## Provenance and peer review

Not commissioned; externally peer reviewed.
